# Ninety years of pulse oximetry: history, current status, and outlook

**DOI:** 10.1117/1.JBO.29.S3.S33307

**Published:** 2024-08-17

**Authors:** Valentina Quaresima, Marco Ferrari, Felix Scholkmann

**Affiliations:** a University of L’Aquila, Department of Life, Health and Environmental Science, L’Aquila, Italy; b University Hospital Zurich, University of Zurich, Biomedical Optics Research Laboratory, Department of Neonatology, Neurophotonics and Biosignal Processing Research Group, Zurich, Switzerland; c University of Bern, Institute of Complementary and Integrative Medicine, Bern, Switzerland; d University of Zurich and ETH Zurich, Neuroscience Center Zurich, Zurich, Switzerland

**Keywords:** pulse oximetry, pulse oximeter, oximetry, CO-oximetry, arterial oxygenation, arterial oxygen saturation

## Abstract

**Significance:**

This year, 2024, marks the 50th anniversary of the invention of pulse oximetry (PO), which was first presented by Takuo Aoyagi, an engineer from the Nihon Kohden Company, at the 13th Conference of the Japanese Society of Medical Electronics and Biological Engineering in Osaka in 1974. His discovery and the development of PO for the non-invasive measurement of peripheral arterial oxygenation represents one of the most significant chapters in the history of medical technology. It resulted from research and development efforts conducted by biochemists, engineers, physicists, physiologists, and physicians since the 1930s.

**Aim:**

The objective of this work was to provide a narrative review of the history, current status, and future prospects of PO.

**Approach:**

A comprehensive review of the literature on oximetry and PO was conducted.

**Results and Conclusions:**

Our historical review examines the development of oximetry in general and PO in particular, tracing the key stages of a long and fascinating story that has unfolded from the first half of the twentieth century to the present day—an exciting journey in which serendipity has intersected with the hard work of key pioneers. This work has been made possible by the contributions of numerous key pioneers, including Kurt Kramer, Karl Matthes, Glenn Millikan, Evgenii M. Kreps, Earl H. Wood, Robert F. Show, Scott A. Wilber, William New, and, above all, Takuo Aoyagi. PO has become an integral part of modern medical care and has proven to be an important tool for physiological monitoring. The COVID-19 pandemic not only highlighted the clinical utility of PO but also revealed some of the problems with the technology. Current research in biomedical optics should address these issues to make the technology even more reliable and accurate. We discuss the necessary innovations in PO and present our thoughts on what the next generation of PO might look like.

## 1 Introduction

The year 2024 marks the 50th anniversary of the invention of pulse oximetry, which was first presented by Takuo Aoyagi, an engineer from the Nihon Kohden Company, at the 13th Conference of the Japanese Society of Medical Electronics and Biological Engineering in Osaka in 1974. The session was chaired by Tatsuo Togawa, a distinguished bioengineer from the Institute of Biomaterials and Bioengineering at Tokyo Medical and Dental University. Takuo Aoyagi’s discovery and subsequent development of optical and pulse oximetry for the non-invasive measurement of peripheral arterial oxygenation represents one of the most significant contributions to the field of medical technology. The development of this technology was the result of a collaborative effort between biochemists, engineers, physicists, physiologists, and physicians, who have been engaged in research and development since the 1930s. Pulse oximetry represents one of the most influential inventions in the field of anesthesia and intensive care medicine, contributing significantly to safety in this field. A multitude of incremental and seminal advances collectively led to the contemporary pulse oximetry, which is currently in use.

In the following, the terms “pulse oximetry” and “pulse oximeter” are abbreviated to “PO” (the specific meaning of each abbreviation will be clear from the context in which it is used).

The aim of this review is to celebrate the 50th anniversary of the disclosure of the invention of PO by summarizing the key stages in the development of optical oximetry in general, and pulse oximetry technology in particular, before and after 1974, up to the recent pandemic which highlighted the shortcomings of PO accuracy and the need for future PO innovations.

The fundamental principles of oximetry, which is the measurement of the proportion of oxygenated hemoglobin (Hb) in the blood, can be traced back to 1864, when the German biochemist Ernst Immanuel Felix Hoppe-Seyler conducted research in Tübingen, Germany, investigating the spectral changes produced by the exposure of crystallized Hb to air.^1^ He therefore employed the spectroscope invented at the same time by Robert Wilhelm Eberhard Bunsen and Gustaf Robert Kirchoff in Heidelberg, Germany.^2^

The invention of PO in 1974 marked the culmination of a period of preliminary work that began in the 1930s in Europe and later in the United States. Currently, PO is a common tool in human medical care, with applications in patient monitoring during surgery, in intensive care units, and in emergency medicine. The advent of the COVID-19 pandemic has rendered the small, affordable finger PO a pervasive tool, comparable in utility to a thermometer, for the non-invasive measurement of peripheral arterial Hb oxygen ($O_{2}$) saturation (${\mathrm{SpO}}_{2}$).^3^ It can be used to easily measure ${\mathrm{SpO}}_{2}$—as an estimate of arterial blood $O_{2}$ saturation (${\mathrm{SaO}}_{2}$)—and heart rate at home with minimal user training. Nevertheless, the global deployment of PO has revealed that the accuracy of PO is influenced by the varying skin pigmentation of individuals. This issue requires immediate attention from manufacturers to ensure the reliability of PO.

Several detailed historical reports have already been published. The first one on oximetry was written by the physiologist Nils Johan Nilsson (University of Lund, Sweden) in 1960.^4^ The most detailed reviews of oximetry and PO were published in the 1980s and 1990s by John Severinghaus (1922–2021) (University of California San Francisco, California, United States) and his collaborators.^5^^–^^12^ Severinghaus was one of the pioneers of anesthesia who, in 1957, developed the first blood gas analyzer to measure the partial pressure of ${\mathrm{CO}}_{2}$ (${\mathrm{pCO}}_{2}$) and the partial pressure of $O_{2}$ (${\mathrm{pO}}_{2}$) in a sample of arterial blood.^13^^,^^14^ A number of comprehensive reviews have provided detailed summaries of the technical principles underlying PO, as well as an analysis of the strengths and limitations of the technology and an assessment of the prospects for further developments.^6^^–^^9^^,^^15^^–^^59^

This review article is intended to provide a further contribution to the existing review articles on the subject, by reporting for the first time some details of various European and Canadian research groups that were active between 1933 and 1974. Furthermore, our article provides a synopsis of the principal technological and regulatory advancements that have occurred over the past three decades, along with an overview of the most recent technological developments in PO.

## 2 Principle of Operation of Pulse Oximetry: A Brief Summary

Prior to a comprehensive historical overview of the development of PO, it is necessary to provide a brief summary of the operating principle of PO.

**Fig. 1:**
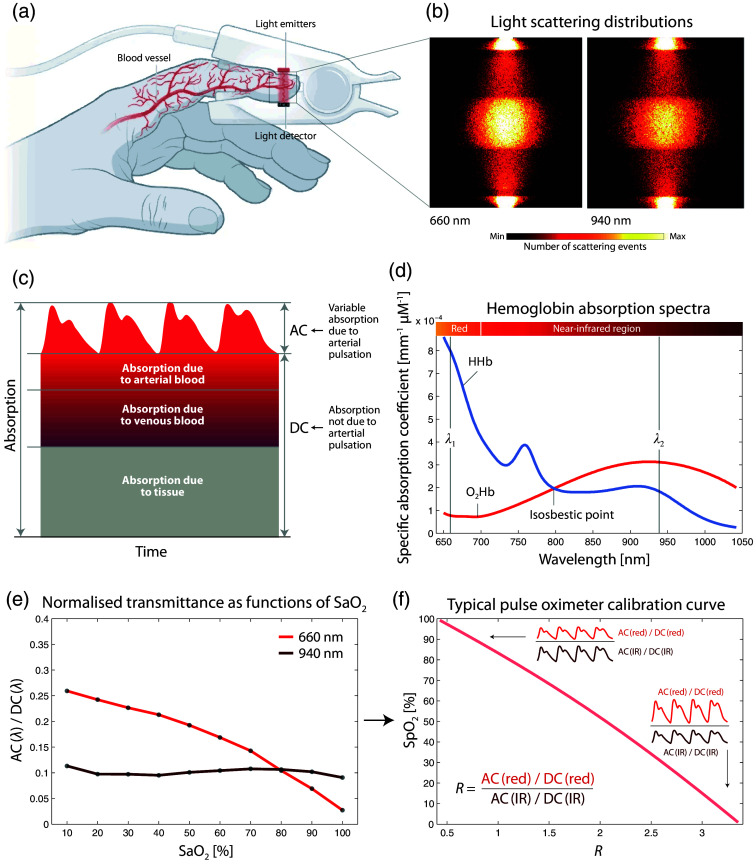
Principle of operation of PO. (a) Representative example of a finger clip of a PO, comprising light detectors and light emitters. Reprinted and modified from Kozlov,^60^ with permission from the publisher. (b) Light scattering distributions in the finger tissue for two wavelengths typically used in PO (red: 660 nm, infrared [IR]: 940 nm). Reprinted and modified from Chatterjee and Kyriacou.^61^ (c) Components of light absorption in PO (visualization redrawn from Moyle^62^). (d) Hb absorption spectra (data source: Biomedical Optics Research Laboratory, UCL, UK). The isosbestic point and two typical PO wavelengths are added. (e) Normalized light transmittance for two wavelengths as functions of ${\mathrm{SaO}}_{2}$ (data: simulation by Chatterjee and Kyriacou^61^). A typical PO calibration curve (data source:^62^) with visualization of the amplitudes of the blood volume pulsation in the two wavelength ranges (red and IR). AC: alternating current (variable signal component), DC: direct current (constant signal component), $O_{2}\mathrm{Hb}$: oxyhemoglobin, HHb: deoxyhemoglobin, ${\mathrm{SaO}}_{2}$: arterial blood oxygenation, ${\mathrm{SpO}}_{2}$: peripheral arterial hemoglobin $O_{2}$ saturation.

The majority of PO devices utilize a finger clip that contains light emitters (typically light-emitting diodes, LEDs) and light detectors (photodiodes) [Fig. 1(a)]. Light with at least two wavelengths is directed through the tissue [see the light scattering distributions shown in Fig. 1(b)] and the transmitted light, which varies in intensity, mainly due to arterial pulsation [Fig. 1(c)], is measured. The wavelengths of the light are selected to enable the absorption changes to the right and left of the isosbestic point of the spectra of oxyhemoglobin ($O_{2}\mathrm{Hb}$) and deoxyhemoglobin (HHb) to be measured [Fig. 1(d)]. The normalized blood volume pulse amplitude is wavelength-dependent on the degree of the $O_{2}$ saturation of Hb (and ${\mathrm{SaO}}_{2}$) [Fig 1(e)]. Consequently, the ratio of the wavelength-dependent blood volume pulsation amplitudes is proportional to ${\mathrm{SaO}}_{2}$; the spectroscopically determined ${\mathrm{SaO}}_{2}$ with the PO technique is then referred to as ${\mathrm{SpO}}_{2}$ [Fig. 1(f)]. The calibration function required for this is determined empirically and depends on the device in question.

It is crucial to be aware of the distinction between functional and fractional Hb saturation.^63^ ${\mathrm{SpO}}_{2}$ measured with PO represents the *functional* Hb saturation (i.e., the ratio between $O_{2}\mathrm{Hb}$ and Hb that is able to carry $O_{2}$, i.e., $O_{2}\mathrm{Hb}$ divided by the sum of $O_{2}\mathrm{Hb}$ and HHb). However, in the blood, there is also a dysfunctional Hb component present, comprising, for example, carboxyhemoglobin (COHb) and methemoglobin (MetHb). When this dysfunctional component is considered, the *fractional* Hb saturation can be determined [i.e., the ratio between $O_{2}\mathrm{Hb}$ and total Hb (i.e., functional + dysfunctional Hb)]. An extended spectral measurement (involving a greater number of wavelengths) enables the concentration of dysfunctional Hb to be determined. When this methodology is employed, it is referred to as CO-oximetry. The devices employed for this purpose are designated as CO-oximeters. It is possible to measure ${\mathrm{SaO}}_{2}$ as either functional or fractional Hb saturation.

## 3 Development of Pulse Oximetry: A Chronological Overview of Its History

The historical development of oximetry and PO in particular is described in detail below, with the development divided into five main phases: 1729–1931, 1932–1971, 1972–1988, 1989–2019, and 2020–2024.

### 3.1 Beginnings of Research Into Cardiopulmonary Physiology

The theory of pulmonary circulation was first proposed in ancient Persia^64^^,^^65^ and subsequently re-established in the thirteenth century by the Egyptian physician Ibn al-Nafis (1213–1288).^66^^,^^67^

From ∼450 to 100 BC, Greek natural scientists (Hippocrates, Herophilius, Erasistratus) also studied cardiovascular physiology and discovered, for instance, that blood flow through the lungs was unidirectional.^68^ Basic anatomical and functional knowledge of the cardiorespiratory system was also gained in Vedic and post-Vedic India.^69^ Published in a theological treatise in 1553, the Spaniard Michael Servetus (1511–1553) (Vienne, Dauphiné, France) is considered the first European eclectic physician to publish a written description of pulmonary circulation, mixing anatomical, and theological terminology.^70^ The fact that the color of the blood changes in the bloodstream was described in his work. The theory of pulmonary circulation was further developed by the anatomist Matteo Realdo Colombo (1516–1559) (University of Padua, Padua, Italy), who proposed that the air is mixed with blood in the lungs. This was subsequently confirmed by the English physiologist William Harvey (1578–1657), who conducted experimental studies in London demonstrating the circulation of blood in organisms and its flow from the veins to the arteries through the heart.^68^^,^^71^ In 1669, the English physician Richard Lower (1631–1691) concluded that the color of the blood reflects the amount of air mixed with the blood.^71^^,^^72^

### 3.2 1729–1931: Insights Into Light Absorption and Spectroscopic Analyses of Blood

Following his investigation into the absorption of light in a transparent body, Pierre Bouguer (1698–1758) (Le Havre Normandy University, Le Havre, France) published his findings in 1729, which demonstrated that the intensity of light decreases in a geometric progression.^73^^,^^74^ In 1760, Johann Heinrich Lambert (1728–1777) (Royal Prussian Academy of Sciences, Berlin, Germany) referenced Bouguer’s discovery and posited that the absorbance of a sample is directly proportional to the path length of the light.^75^ In 1852, August Beer (1825–1863) (University of Bonn, Bonn, Germany) demonstrated that the absorbance is proportional to the concentration of the absorbing substance.^76^ A few years later, the physicist Gustav Robert Kirchoff (1824–1887) and the chemist Robert Wilhelm Eberhard von Bunsen (1811–1899) (University of Heidelberg, Heidelberg, Germany) employed their flame spectroscope to identify the spectral lines of each element, thereby establishing the science of spectroscopic analysis as a tool for analyzing the composition of matter.^2^

The pigment responsible for the red color of the blood was crystallized from various animals as early as the 1840s.^77^ In 1862, the biochemist Felix Hoppe-Seyler (1825–1895) (University of Tübingen, Tübingen, Germany) published his findings on the spectroscopic properties of the previously unidentified “Blutfarbstoff” (“blood pigment”). His research concentrated on the pigment’s capacity to absorb light, irrespective of the various color changes that occur in the blood due to the presence of $O_{2}$, ${\mathrm{CO}}_{2}$, carbonic acid, hydrogen arsenide, and other substances.^78^ In 1864, Felix Hoppe-Seyler conducted an experiment investigating the crystallization of blood in the presence and absence of $O_{2}$.^1^ In the same year, George Gabriel Stokes, an Irish physicist and mathematician (1819–1903) (University of Cambridge, Cambridge, United Kingdom), was the first to demonstrate spectroscopically the respiratory function of Hb (or “cruorine,” as he himself called it).^79^ The observed spectral changes were attributed to the effect of air on Hb solutions. Hb exists in two states, which are distinguished by a visible color difference (red for the oxygenated state and deep red/purple for the deoxygenated state). Stokes designated these two substances as scarlet “cruorine” and purple “bruorine.” However, Hoppe-Seyler proposed “oxyhemoglobin” and “deoxyhemoglobin,” which are the names currently in use.

The first *in vivo* demonstration of these spectral differences was conducted by physiologist Karl von Vierordt (1818–1884) (University of Tübingen, Tübingen, Germany), who published his observations of spectroscopic measurements of blood in 1876.^80^

**Fig. 2:**
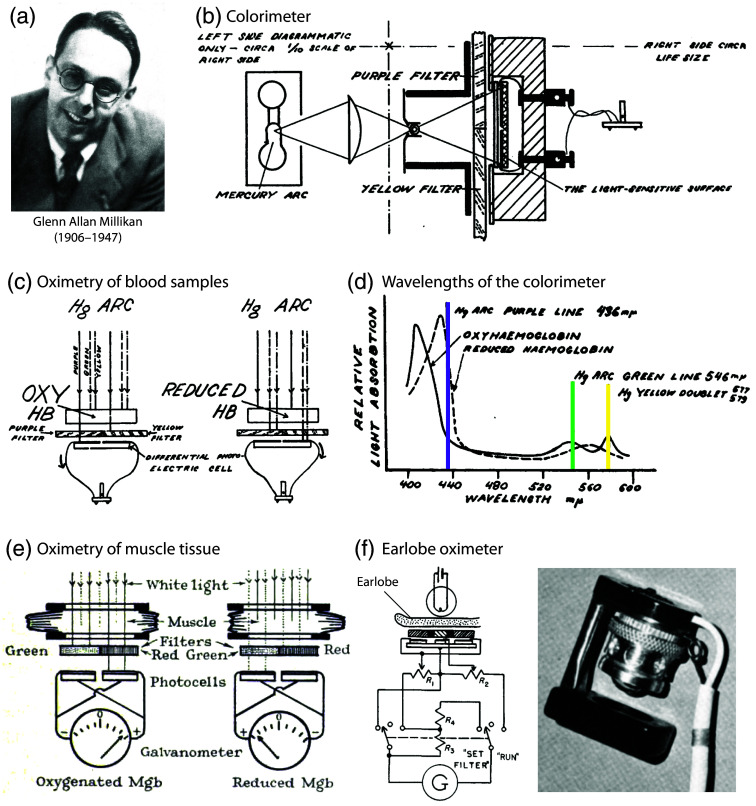
Spectrophotometric devices from the 1930s–1950s developed by Glenn Allan Millikan to measure blood oxygenation. (a) Picture of G. A. Millikan. Reprinted from Millikan,^81^ with permission from the publisher. (b, c) Principle of operation of the invented photoelectric colorimeter for oximetry of blood samples described in a publication in 1933.^82^ (d) Wavelengths used by the colorimeter. (b)–(d) Reprinted and modified from Millikan,^82^ with permission from the publisher. (e) A spectrophotometric oximeter developed by Millikan to perform muscle measurements on living animals.^83^ Reprinted from Millikan,^83^ with permission from the publisher. (f) Circuitry and picture of a spectrophotometric earlobe oximeter developed by Millikan and described in a publication in 1942.^84^ Circuitry visualization reprinted and modified from Millikan,^84^ with permission from the publisher. Picture of the Millikan’s oximeter (right) taken from Wood,^85^ with permission from the publisher.

In the late 1920s, Glenn Allan Millikan (1906–1947) [Fig. 2(a)], an American physiologist, constructed a two-wavelength photoelectric colorimeter to quantify the oxygenation of Hb solutions during his doctoral studies in Cambridge, United Kingdom, where he worked until 1938. Millikan was the second son of Robert Andrew Millikan, an American experimental physicist who was awarded the Nobel Prize in Physics in 1923 for demonstrating the quantization of electric charge and for his work on the photoelectric effect. Glenn Millikan received his education at various institutions, including the University of Chicago, Harvard University, several German universities, and finally at Cambridge University.

His supervisor at Cambridge was Lord Adrian (1889–1977), a professor of physiology, and the recipient of the 1932 Nobel Prize in physiology. Millikan is renowned for his investigations into the rate of reaction of $O_{2}$ with Hb and myoglobin. In the Millikan photoelectric colorimeter, the light from a mercury arc lamp was passed through purple and yellow filters, allowing the detection of a distinct change in light intensity as it passed from one end of the color spectrum to the other. This change was measured on one side of the differential photoelectric cell, which was made of copper oxide. Absorbance values for oxygenated and deoxygenated Hb were determined, and calibration curves were derived. The device exhibited an overall accuracy of ∼4%. In 1933, Millikan published the invention of his photoelectric colorimeter.^82^ For a visualization of the operation principle of the device, please refer to Figs. 2(b)–2(d).

It is also pertinent to mention that Britton Chance (1913–2010) (University of Pennsylvania, Philadelphia, Pennsylvania, United States), the founder of biomedical photonics and the founder of the *Journal of Biomedical Optics* in 1996, commenced his second PhD in physiology at Cambridge under the tutelage of the physiologists Hamilton Hartridge (1886–1976), Francis Roughton (1899–1972), and Millikan, who was a postdoctoral fellow. In 1937, Chance became Millikan’s research student and collaborated with him on the development of an innovative stop-flow apparatus. In the same year, Millikan developed a spectrophotometric oximeter to measure muscle oxygenation in living animals^83^ [Fig. 2(e)].

In 1931, Bruno Albert Lange (1903–1969), a German physicist, published the discovery of the photoelectric effect in selenium barrier-layer cells.^86^ Lange conducted experiments with the selenium photocell at the Kaiser Wilhelm Institute for Silicate Research in Berlin. The photocell was capable of converting light into sufficient electrical energy to operate a motor. Selenium barrier-layer cells, manufactured by the company Dr. Bruno Lange GmbH (Düsseldorf, Germany), have been the dominant technology in oximetry since their introduction. Initially, they were used in Germany and, subsequently, in Europe and the United States. This dominance is largely attributable to the ability to construct these cells in a very small size. It is also notable that in the same year, 1938, Ludwig Bergmann (1898–1959), a German physicist (University of Wrocław), independently published the development of a selenium barrier-layer photocell.^87^ As Bergmann pointed out in a published commentary, Lange failed to cite his work in his publication.^88^

### 3.3 1932–1971: The First Non-Invasive O_2_ Saturation Measurements Before the Discovery of Pulse Oximetry

**Fig. 3:**
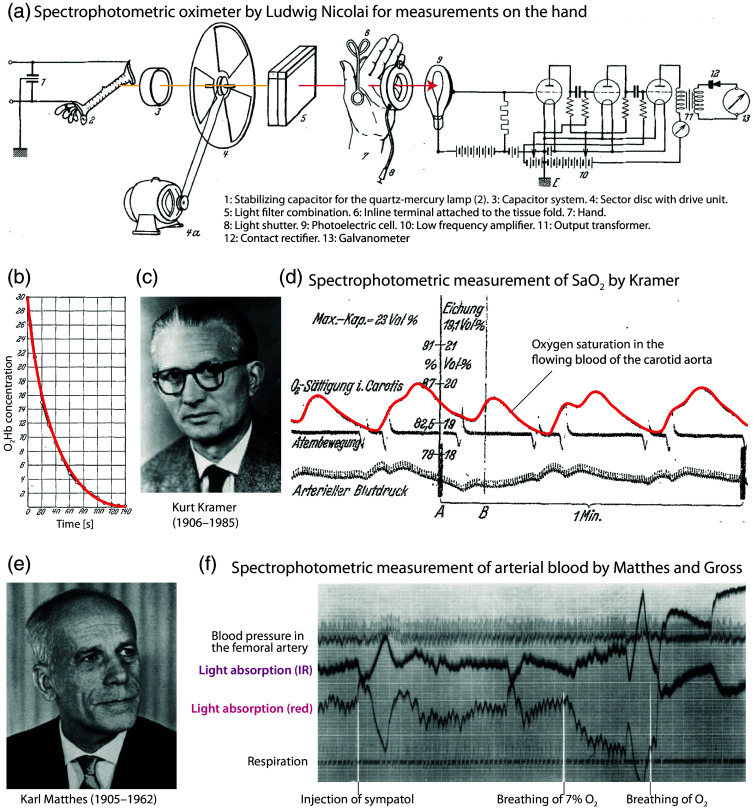
Spectrophotometric devices and measurements from the 1930s developed and performed by Ludwig Nicolai, Kurt Kramer, and Karl Matthes. (a) Experimental setup developed by Nicolai to measure Hb oxygenation non-invasively on the hand.^89^ The light path is marked in red. (b) Changes in $O_{2}\mathrm{Hb}$ after arterial occlusion on the arm. Figures (a) and (b) reprinted and modified from Nicolai,^89^ with permission from the publisher. (c) Picture of Kurt Kramer. Reprinted from the professor catalog of the University of Leipzig, with permission from the University of Leipzig, Germany. (d) Changes of $O_{2}$ saturation in the flowing blood of the carotid aorta of a dog were measured with the method of Kramer.^90^ Reprinted and modified from Kramer,^90^ with permission from the publisher. (e) Picture of Kurt Matthes. Reprinted from von Kress,^91^ with permission from the publisher. (f) Example of spectrophotometric measurements of Matthes and Gross on a dog.^92^ Reprinted and modified from Matthes and Gross,^92^ with permission from the publisher.

In 1932 (i.e., 55 years after the first *in vivo* measurements by von Vierordt), Ludwig Nicolai (1904–unknown date of death), an Austrian physicist working at the University of Göttingen, Germany, published his findings on the decay of oxygenated Hb in the tissue of the hand following the occlusion of the wrist circulation^89^ [Fig. 3(a)]. He alternated the wavelengths of the blue-green light emitted by a mercury-arc lamp, which was invented in 1901 by the American engineer Peter Cooper Hewitt (1861–1921), and utilized the photoelectric effect of the selenium barrier-layer cells of Bruno Lange GmbH. Nicolai observed that the concentration of oxygenated Hb decreased exponentially during occlusion [Fig. 3(b)].

From 1933, Kurt Ludwig Heinrich Kramer (1906–1985) [Fig. 3(c)], a PhD student in Nicolai’s laboratory, accurately recorded variations in oxygenated Hb based on $O_{2}$-dependent Hb spectral differences by passing monochromatic red light through hemolyzed blood or, for the first time, through blood flowing in large arteries (in the dog)^90^^,^^93^^–^^95^ [Fig. 3(d)]. This can be considered the first instrument for the continuous optical measurement of $O_{2}$ saturation. Kramer replaced Nicolai and Millikan’s light source with an ordinary light bulb with a Zeiss red filter; Bruno Lange’s selenium barrier-layer photocells served as the detector. The method was presented to the Medical Society in Göttingen on November 3, 1933. The Hb saturation was measured in closed vessels of animals with an accuracy of ±1% in comparison to Van Slyke’s analyses. Donald Dexter Van Slyke (1883–1971) was a Dutch-American biochemist who invented the volumetric gas apparatus for measuring and calculating ${\mathrm{CO}}_{2}$, $O_{2}$, and ${\mathrm{HCO}}_{3}^{-}$ in the blood, the first apparatus designed specifically for the clinical chemistry laboratory. The detailed description of the Kramer method was published in an article in 1934.^90^

The other German scientist who independently contributed to the development of oximetry before the Second World War was Karl Matthes (1905–1962) [Fig. 3(e)]. In 1934, at the University of Leipzig, Germany, Matthes developed a similar device for measuring blood oxygenation in intact vessels. Subsequently, the two German pioneers employed the method on intact tissues, including the earlobe and auricle.^96^ In 1935, using a mercury arc lamp and a red-sensitive photocell used by Kramer, Matthes was the first to use two spectral ranges selected by red and green filters, one of which was unaffected by $O_{2}$, to compensate for changes in tissue thickness, blood content, light intensity, and other variables.^97^ In 1938, Karl Matthes and Franz Gross developed the first oximeter system that used two spectral ranges, as is done in today’s POs^92^ [Fig. 3(f)].

The oximeter was used to measure the Hb saturation trends of the transilluminated earlobe after histamine iontophoresis. The other histaminized earlobe was used to obtain a plethysmogram, which was used to assess passive changes in the vascular bed. The device was a red-infrared ear $O_{2}$ saturation meter. The infrared detector was a gas-filled phototube, covered with a filter that allowed only infrared light to pass through. The bulky device was slow, required frequent calibration, and was inconvenient to use. Matthes’ device was ingenious, yet it was unable to provide absolute saturation values. Nevertheless, it is considered to be the first $O_{2}$ saturation meter. It is noteworthy that from October 1929 to September 1931, Matthes worked in London at the National Institute for Medical Research and subsequently at the Physiological Institute in Oxford, where he collaborated with the future Nobel Prize winner for medicine Charles Scott Sherrington (1857–1952). Unfortunately, Matthes was the victim of racial laws due to his wife’s ancestry. Furthermore, in January 1945, he was threatened with deportation to a labor camp. Matthes has been designated the “father” of oximetry by John Wendel Severinghaus, Earl Howard Wood, and others. Nevertheless, Kramer was engaged in continuous research for at least 23 years, from 1933 to 1956. His work involved the development and testing of new methods for the non-invasive measurement of Hb oxygenation in experimental animal models and in humans. Initially, this was conducted in Germany and the United Kingdom prior to the Second World War and subsequently in the United States following the war. Accordingly, it seems reasonable to conclude that both men should be regarded as the “fathers” of oximetry.

In 1932, Joseph Barcroft (1872–1947) began studying the physiology of the developing fetus at the Laboratory of Physiology at the University of Cambridge, United Kingdom. Prior to his habilitation in physiology at the University of Heidelberg, Germany, in 1937, Kramer worked with Millikan in Barcroft’s laboratory in 1935. During birth by cesarean section, continuous measurements of the ${\mathrm{SaO}}_{2}$ value in the carotid blood of lambs were carried out with an oximeter using the Bruno Lange selenium-barrier photocells, which Kramer had brought to Cambridge. The data were collected in the years 1935–1936 and published in 1939.^98^

In 1937, the physiologist Alrick B. Hertzman (1898–1991) first observed the “photoplethysmogram” at St. Louis Uiversity (St. Louis, Missouri, United States) and coined the term “photoelectric plethysmograph,” as the technique was based on the use of a polychromatic light source and a photocell to observe the relationship between the backscattered light and the volumetric changes in skin blood.^99^ This technique was later called photoplethysmography (PPG). As early as 1934, the pharmacologist M.R. Bonsmann (Leipzig, Germany) used photocells to measure the blood pressure in the tail of rats.^100^ Although PPG was considered an interesting tool, the PPG waveform was never intensively studied. It was not until the discovery of PO in 1974 that it gained significant importance. The PPG waveform is commonly displayed in clinical PO devices.^101^

**Fig. 4:**
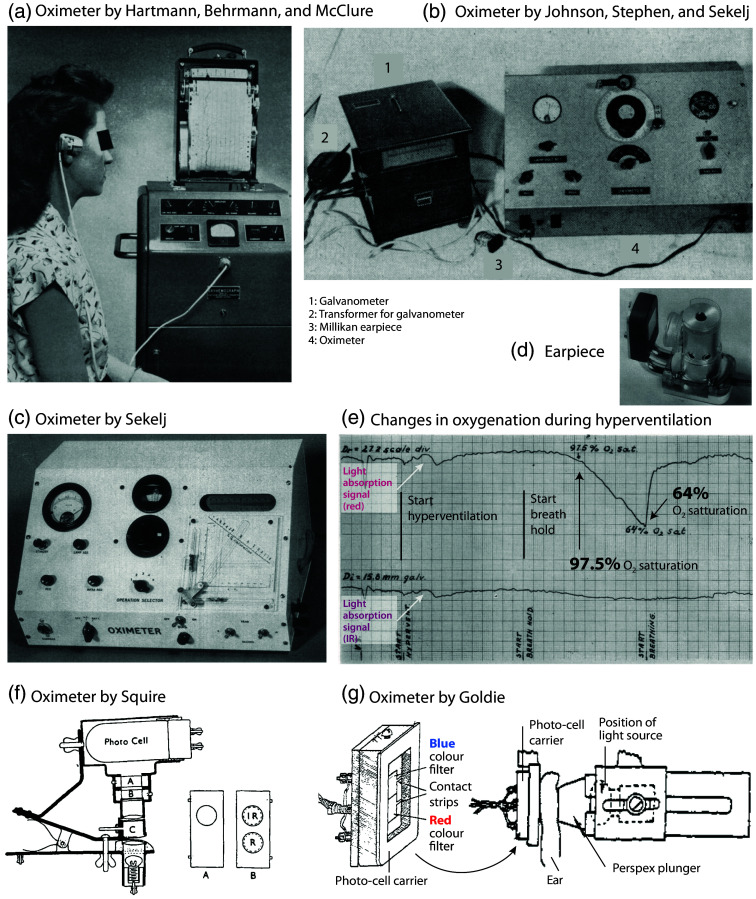
Spectrophotometric oximeters from the 1940s–1950s developed by the groups of Hartmann, Sekelj, Squire, and Goldie. (a) Oximeter with earlobe probe (“oxyhemograph”) developed by Hartmann, Behrmann, and McClure^104^ and manufactured by Photocon Research Products (Pasadena, California, United States). Reprinted from Hartmann et al.,^104^ with permission from the publisher. (b) Oximeter developed by Johnson, Stephen, and Sekelj.^105^ Reprinted from Johnson et al.,^105^ with permission from the publisher. (c) Oximeter developed by Sekelj with a new earpiece (d) with improved optical characteristics for better accuracy. (e) Measurement example (deoxygenation due to hyperventilation followed by breath-hold in an adult) with the device shown in panel (c). Panels (c), (d), and (e) reprinted and modified from Sekelj,^106^ with permission from the publisher. (f) Oximeter developed by Squire for measurements on the hand skin fold.^107^ The capsule (C) was pressed onto the skin to create a blood-free tissue condition. Two sliders were used, one to set the zero point (A), and the other to filter the light (red and IR) (B). Reprinted from Squire,^107^ with permission from the publisher. (g) Oximeter developed by Goldie^108^ for measurement of the ear. For the measurement, the blood was expelled from the ear by being squeezed by the glass plate and the plunger. Reprinted and modified from Goldie,^108^ with permission from the publisher.

In 1939, Frank W. Hartman (1890–1979) and Roy D. McClure (1882–1951) of the Henry Ford Hospital in Chicago (Illinois, United States) published an article on the results of investigations conducted using a newly developed single wavelength hand oximeter operating in the red region.^102^ This “oxyhemoglobinograph” comprised a photoelectric colorimeter in which the light beam passed through the tissue of the hand, which was heated by the photoelectric cell. A revised version, incorporating an ear clip, was published in 1948.^103^ A year later, the authors presented a further development of the apparatus (“oxyhemograph”), which was then manufactured by Photocon Research Products (Pasadena, California, United States)^104^ [Fig. 4(a)].

As previously stated, the Matthes ear $O_{2}$ saturation oximeter presented significant challenges when attempting to calibrate it. In the late 1930s, John Rupert Squire (1915–1966), a medical student at the University College Hospital Medical School of London, United Kingdom, constructed a self-calibrating oximeter for measurements on the hand. Additionally, he proposed that the relative signal strengths of red and infrared light should be adjusted to be equal when blood flow is interrupted by tissue compression^107^ [Fig. 4(f)]. Squire discovered that when the red and infrared light passing through tissue was measured before and after the blood was squeezed out of the interdigital space of the hand by pneumatic pressure, the logarithm of the ratio of the ratios of these four transmitted light intensities (red and infrared when perfused and when blanched) was a function of ${\mathrm{SaO}}_{2}$.

The Second World War prompted the necessity for an oximeter that could be utilized in unpressurized cabins of military aircraft, as fighter pilots were prone to fainting under high pressure. At the onset of the war, the inaugural objective of the newly established Royal Air Force (RAF) Physiological Laboratory at Farnborough, Hampshire, United Kingdom, was to identify a solution to the issue of how to supply $O_{2}$ to aircrews in a more efficient and economical manner. Physiologist E.A.G. Goldie developed a heated ear oximeter and designated the “anoxia meter”^108^ [Fig. 4(g)]. The instrument employed a battery-operated torch bulb and photocells (manufactured in the United Kingdom by Messrs. Electrophysical Laboratories Ltd. and by Salford Electrical Instruments Ltd.) covered by a suitable color filter. The device, which was published in an article in 1942, was capable of taking standardized measurements by shining a light through the ear and squeezing out blood between the glass plate and the plunger. The device exhibited an accuracy of ∼2%. Although larger than the oximeter developed by Millikan in Philadelphia (Pennsylvania, United States) in the same period, the “anoxia meter” proved invaluable in evaluating the relative merits of different types of $O_{2}$ masks and revolutionized the study of anoxia by enabling the estimation of the amount of $O_{2}$ in the blood non-invasively at any time and for any length of time.

In the spring of 1940, Lord Adrian (1889–1977), Millikan’s former Cambridge PhD supervisor, requested his assistance in developing a breathing apparatus to prevent fighter pilots from losing consciousness under high gravitational forces. It was only after the United States military learned that the German Air Force (Luftwaffe, Bonn, Germany) was equipped with oximeters and that a hypoxia warning device allowed their pilots to fly safely at higher altitudes that they became interested in Millikan’s research and development of the oximeter. In the period between 1940 and 1941, Millikan collaborated with the Bendix Company (Avon, Ohio, United States) to develop an in-flight $O_{2}$ supply system.^109^ During the Second World War, Bendix manufactured a range of equipment for military aircraft, including radio transceivers and radar equipment. The pilot’s blood $O_{2}$ status was monitored by a sensor, the earlobe oximeter, which was integrated into the pilot’s mask. The oximeter was employed as a feedback loop. The initial data were presented at the meeting of the American Physiological Society in 1941.

From 1940 to 1942, Millikan served as the director of the Eldridge Reeves Johnson Foundation for Research in Medical Physics, which was established in 1929 at the University of Pennsylvania School of Medicine (Philadelphia, Pennsylvania, United States). In collaboration with John Pappenheimer (1915–2007) (Harvard University, Cambridge, Massachusetts, United States), he constructed a lightweight heated ear oximeter for use in aviation research during pilot training and to monitor $O_{2}$ delivery in pilots^84^^,^^110^ [Fig. 2(f)].

Millikan is credited with coining the term “oximeter.” Millikan drew upon two German concepts: the contributions of Kramer, who developed copper oxide barrier photocells, and Matthes, who devised red and green filters, were also significant. The instrument employed a battery-powered light source and red and green filters to provide two different wavelengths. Millikan postulated that the light transmitted through the green filter was independent of ${\mathrm{SaO}}_{2}$. However, he subsequently discovered that the ear is opaque to green light and that the transmitted light could be measured in the near-infrared spectral region. The oximeter was a relative reading instrument that required a known or arbitrary starting point for calibration. The oximeter reading was used to regulate the supply of $O_{2}$ to the pilot’s mask, which was constructed with an oximeter integrated into it. It is of interest to note that Britton Chance commenced his employment at the Johnson Research Foundation in 1940 under the direction of Millikan.^111^^,^^112^ Chance was the director of this foundation from 1949 to 1983.

The Millikan oximeter was used extensively in the study and control of hypoxemia induced by breathing gas mixtures with low $O_{2}$ or in a low-pressure chamber.^113^ The oximeter, designated the “anoxia photometer” Model 17A, was manufactured by the Coleman Electric Company (New York, United States) in 1948 and subjected to testing by the Aero Medical Research Unit at Wright Field (Welfare Island, New York, United States) and by the Cardiology Department, Columbia University College of Physicians and Surgeons (New York, New York, United States). The oximeter was unsuitable for use in aircraft due to the necessity of a stable galvanometer to accommodate the low current signals. It is noteworthy that the Millikan oximeter was employed in 1946 at the Medical Division, Edgewood Arsenal, Aberdeen Proving Ground (Maryland, United States) to ascertain the circulation time during breath holding and a single deep breath of 100% nitrogen.^114^

Following the conclusion of the Second World War and until the end of the 1950s, a number of research groups in the United States, Canada, and Europe independently replicated and/or improved upon Millikan’s oximeter. The most significant efforts were made in the United States, initially by Kramer. Kramer, who was an officer in the Schutzstaffel (SS), Adolf Hitler’s paramilitary organization, developed an oximeter for the German Air Force (Luftwaffe) during the war. At the conclusion of the World War II, Kramer was held as a prisoner of war in Germany for 18 months by the Soviet Union and subsequently by the United States. Following his release from captivity, he was employed at the US Air Force School of Aviation Medicine (Randolph Air Force Base, Texas, United States) until 1950 as part of the “Operation Paperclip,” a top-secret U.S intelligence program that brought Nazi German scientists to America to utilize their expertise for Cold War initiatives.^115^ During this period, Kramer developed photoelectric methods for determining $O_{2}$ saturation, Hb concentration, and dyes in unopened vessels. In addition, he designed and tested a photoelectric hypoxia warning device for high-altitude environments, capable of detecting low blood $O_{2}$ levels and alerting the user. An alarm signal was generated when the ${\mathrm{SaO}}_{2}$ level fell below 85%, with a time to alarm of ∼40  s.

In 1946, at Washington University of St. Louis (Missouri, United States), Kramer assisted James Otis Elam (1918–1995) and Kenneth Sugioka (1920–2014) in the construction of a dual-wavelength ear oximeter for the US Army Air Force. This device employed selenium junction photocells and Wratten filters.^116^ In 1949 and 1951, Kramer and Elam published two articles in which they identified several sources of error in optical oximetry that were attributable to the inadequacy of the Beer-Lambert law.^117^^,^^118^ It is noteworthy that Elam initiated the development of contemporary techniques for cardiopulmonary resuscitation, including the rescue breathing method.

**Fig. 5:**
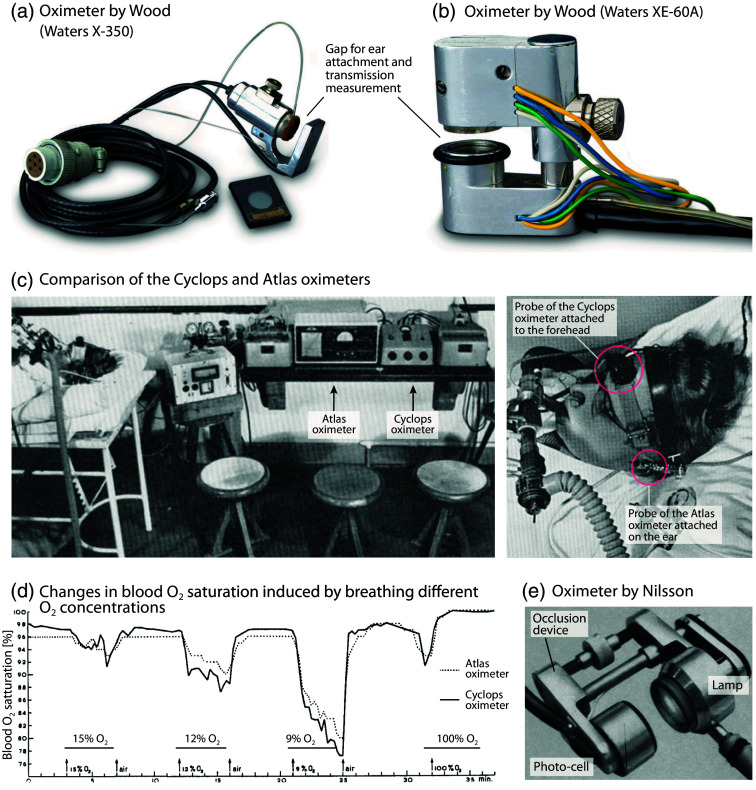
Spectrophotometric oximetry devices from the 1950s. (a), (b) Wood oximeters Waters X-350 and Waters XE-60A for oximetry measurements on the ear. Image courtesy of the Wood Library-Museum of Anesthesiology, Schaumburg, Illinois, United States. (c), (d) Comparison of the Cyclops and Atlas oximeters by measuring in transmission mode at the ear (Atlas; transmission mode) and on the forehead (Cyclops; reflectance mode) on a subject while breathing different $O_{2}$ concentrations.^123^ Figures (c) and (d) reprinted and modified from Zijlstra et al.,^123^ with permission from the publisher. (e) Ear oximeter developed by Nilsson.^124^ Reprinted and modified from Nilsson,^124^ with permission from the publisher.

In 1948, McClure et al. (Henry Ford Hospital, Chicago, Illinois, United States), in collaboration with General Motors Laboratories, employed an enhanced ear “oxyhemograph” comprising dichromatic photocells in a multitude of surgical procedures necessitating anesthesia of between 30 min and 10 h duration.^119^ In 1948, Earl Howard Wood (1912–2009), J. E. Geraci, and colleagues at the Mayo Clinic in Rochester (Minnesota, United States) initiated the development of a self-calibrating, continuous-reading ear oximeter.^120^^–^^122^ Millikan’s ear oximeter was redesigned with a tungsten bulb light source, a second galvanometer, an improved infrared filter, and the addition of an inflatable balloon. The balloon was used to stop the blood flow (compression around 200 mmHg) and obtain a calibration (initial zero) that considered the tissue to be bloodless. Once the pressure was released and the blood was allowed to reperfuse, the difference between the initial zero and the peak was measured, thereby providing the actual blood $O_{2}$ saturation (with an accuracy of ∼3%). Wood published the fundamental equations that underpin the system’s operation. The oximeter was patented by Wood and assigned on April 26, 1955. The oximeter, manufactured by Waters Conley Co. (Minneapolis, Minnesota, United States), was employed in a variety of contexts until 1950, including exercise testing, neonates, thoracic surgery, aviation, and numerous other experimental settings. However, it was rarely used to monitor $O_{2}$ saturation during anesthesia or intensive care. More than 60 articles were published prior to 1950. The oximeter was capable of compensating for the differences in melanin content between light-skinned and darker-skinned individuals; however, it was not reliable due to the instability of the light source and the selenium barrier-layer photocells. The oximeter was particularly susceptible to damage and cumbersome to use. Waters Conley Co.’s successor, Waters Company (Rochester, Minnesota, United States), continued to manufacture commercial oximeters. In 1960, Waters introduced the Model XE-60A ear oximeter [Fig. 5(b)] and subsequently the Waters XP-350 [Fig. 5(a)] and Waters 0-1100. These devices were similarly cumbersome, difficult to calibrate, sometimes nonlinear, and used uncomfortable ear probes that were difficult to apply. In 1952, John F. Perkins (1909–1966) and colleagues at the University of Chicago (Chicago, Illinois, United States) converted the Millikan and Wood oximeters into direct-writing recorders for clinical use.^125^

In 1950, W. Paul, a researcher at the University of Toronto (Ontario, Canada), developed a direct-writing ear oximeter. This device employed an incandescent lamp, red and infrared filters, two selenium barrier-layer photocells, and a pressure capsule.^126^^–^^128^ In 1950, P. Sekelj, C. R. Stephen, and colleagues at McGill University of Montreal (Québec, Canada), in collaboration with the Children’s Memorial Hospital, published a modified Millikan ear oximeter for use in the operating theatre^105^^,^^129^ [Fig. 4(b)]. For the first time, there were reports of frequent and significant intraoperative desaturations. The instrument required frequent calibration, was delicate, and sometimes burned the patient’s ear. A further improvement was published in 1954, which included a new earpiece and a new graphical calculation device for determining the $O_{2}$ saturation^106^ [Figs. 4(c)–4(e)]. In South Africa, engineer Cecil S. Paterson constructed a modified Wood ear oximeter for anesthesia monitoring in 1951. The oximeter was described in an article published in 1952.^130^

After the war, numerous academic laboratories and companies were engaged in research activities across Europe. In Sweden, the engineer K. G. Berg constructed an oximeter based on the work of Millikan in 1947, which was utilized in clinical settings for a period of 2 years at the 4th Medical Service of St. Erik Hospital in Stockholm. In 1948, Inga Lindgren from this hospital reported the continuous measurement of ${\mathrm{SaO}}_{2}$ with this device.^131^ In 1949, Robert Brinkman (1894–1994) and Willem Gerrit Zijlstra (1931–1979) from the University of Groningen, the Netherlands, published their development of the first forehead oximeter, which employed a selenium barrier-layer photocell.^132^ The oximeter was a relative reading instrument that required a known or arbitrary starting point to be set. The “Cyclops” forehead reflexometer from P. J. Kipp and Sons (Delft, The Netherlands) [Figs. 5(c), 5(d)] was not straightforward to use. It was the precursor to a number of reflectance oximeters used for the measurement of blood *in vivo* and *in vitro*.

In 1951, Albert Alois Bühlmann (1923–1994), a distinguished physiologist at the University Hospital of Zurich, Switzerland, who had made significant contributions to the fields of high-altitude and high-pressure respiratory physiology, constructed an advanced dual-wavelength reflectance oximeter that was independent of Hb concentration.^133^^,^^134^ From 1951 onwards, the German company Atlas Werke (Bremen) developed the ear oximeter, designated the “Atlas Universal Oximeter” [Figs. 5(c), 5(d)]. The sensor incorporated Zeiss interference filters (625 and 810 nm) and a selenium photodetector. The auricle was compressed by a screw bushing, and the pressure was regulated by a manometer. The instrument exhibited an accuracy of 1% within the range of 40% to 100%.

In 1956, Nils Johan Nilsson of the Physiological Institute of the University of Göttingen, Germany, presented a new oximeter designed for the measurement of $O_{2}$ saturation in the blood, with a particular focus on the ear and flow-through cuvettes^124^ [Fig. 5(e)].

In the Soviet Union in the early 1950s or even earlier, the physiologist and biochemist Evgenii Mikhailovich Kreps (1899–1985), the engineer M. S. Shipalov, and their colleagues developed in St. Petersburg the dual-wavelength ear oximeters, designated as the “oxyhemometer” and the “oxyhemograph.” These devices were also developed in the context of the advancement of space travel.^135^^–^^138^ It was possible to equip the “oxyhemometer” with a miniaturized radio telemetry device for use in conjunction with a spaceflight helmet. From 1958 onward, L. F. Sochivko and colleagues at the Biofizpribor Company (St. Petersburg) developed oximeters designated OP-01 and POG-01, which were capable of detecting the $O_{2}$ saturation of Hb in arterial and venous blood.^137^^,^^139^^,^^140^ In the 1960s, Krasnogvardeets (Leningrad), the largest medical equipment factory in the Soviet Union, commercialized the O26, O36, and O57 ear oximeters with two photodetectors (selenium and silver sulfide). These were used in aviation, clinical practice, diving physiology, and sports medicine until the late 1980s.^137^

In former Czechoslovakia, an ear oximeter was constructed and employed during surgical anesthesia, with the results published in 1955.^141^

**Fig. 6:**
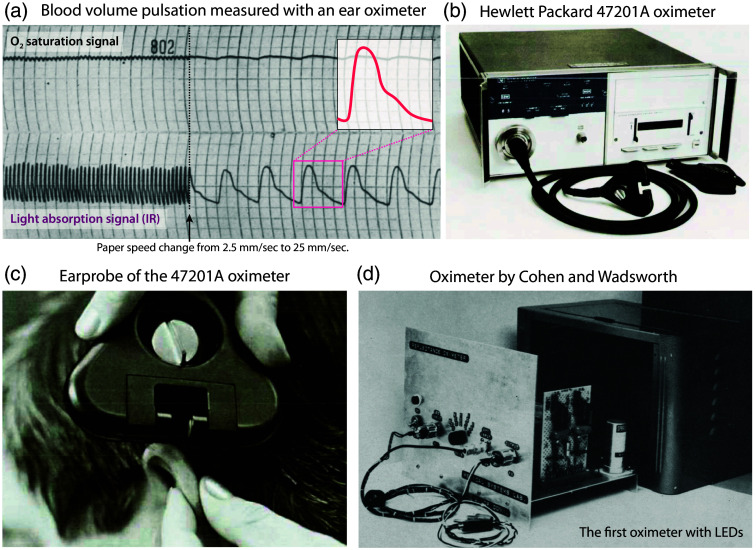
The first detailed measurements of the blood volume pulsation with an oximeter and oximeter devices from the 1970s. (a) Schotz et al. documented the exact waveform of the blood volume pulse with oximeter measurements between 1958 and 1960 and demonstrated the physiological usefulness of this signal characteristic.^142^^–^^144^ Reprinted and modified from Schotz et al.,^144^ with permission from the publisher. (b), (c) Hewlett-Packard’s 47201A oximeter. Reprinted from Merrick,^145^ with permission from the publisher. (d) Skin reflectance oximeter developed by Cohen and Wadsworth; the first oximeter with light emitting diodes (LEDs).^146^ Reprinted from Cohen and Wadsworth,^146^ with permission from the publisher.

In the United States, in 1958, Seymour Schotz and colleagues (Thomas Jefferson University, Philadelphia, Pennsylvania, United States) demonstrated that ear oximetry is a sensitive circulatory monitoring tool that is highly valuable in assessing cardiac output.^142^^–^^144^ A pulsatile trace, a wave-like trace associated with pulsatile blood flow to the ear, was observed in the near-infrared data from the Wood ear oximeter combined with an electronic recorder (ink-writing polygraph at a paper speed of 25  mm/s; Grass Instrument Company, Quincy, Massachusetts, United States) [Fig. 6(a)]. The near-infrared blood volume pulse was identified as an appropriate signal for evaluating alterations in cardiac stroke volume output.

In 1959, at Indiana University School of Medicine (Indianapolis, Indiana, United States), John B. Hickam (1915–1970), Herbert O. Sieker (1924–2016), and Regina Frayser developed a two-wavelength retinal vascular oximeter and reported the first measurement of blood $O_{2}$ saturation in human retinal vessels.^147^^,^^148^ Since 1962, fiber optic catheters have been employed for intravascular and intracardiac reflection oximetry. In 1963, a portable wireless oximeter with interferential filters was developed by NASA’s Ames Research Center (the primary NASA research facility located at Moffett Federal Airfield, Mountain View, California, United States) in collaboration with Beckman Instruments (Brea, California, United States). The development of the telemetry system was not entirely successful. It was employed solely in the centrifuge and was never utilized on space missions. In 1972, the US Air Force School of Aerospace Medicine (San Antonio, Texas, United States) developed two dual-wavelength oximeters (640 and 790 or 805 nm). However, these were never used in flight due to their susceptibility to motion-induced interference.

The first International Colloquium on oximetry was organized by Kramer in Bremen (Germany) in January 1959. In the following year, the proceedings of this meeting were published.^149^ In the same year, the physiologist Nils J. Nilsson (University of Lund, Lund, Sweden), who had worked with Kramer at the University of Göttingen (Göttingen, Germany) in the 1950s, published the first detailed review on oximetry.^4^ The review encompassed a total of 260 references, with 30 and 12 articles, respectively, featuring Kramer and Matthes as the first authors. It is probable that this review did not encompass the research activities of Soviet scientists, as Nilsson was unable to access their articles published in Russian journals.

Prior to the introduction of PO in 1974, calibration represented a significant challenge in oximetry. The method of calibration by compressing the tissue until it is blood-free, as employed by Squire on the interdigital space and subsequently by Goldie and Wood on the ear, was found to have the disadvantage of being time-consuming and requiring individual calculation.^4^ Furthermore, the method employed was not sufficiently accurate, as it was not possible to obtain truly blood-free tissue. The highest degree of accuracy (2.9%) was obtained with the oximeter of Wood in comparison to the Van Slyke measurement. In conclusion, all oximeters constructed prior to Aoyagi’s discovery exhibited a deficiency in calibration, rendering them incapable of providing absolute ${\mathrm{SaO}}_{2}$ values. Instead, they were only useful for measuring changes.

In 1962, Y. Enson and colleagues (Columbia University College of Physicians and Surgeons and Bellevue Hospital, New York, United States) published an article describing the development of a reflection oximeter that used two bundles of flexible glass fibers and could measure blood oxygenation at the tip of an arterial needle or a cardiac catheter.^150^ The device demonstrated an accuracy of 1.9% and was successfully employed in the diagnosis of patients suspected of having congenital heart disease.^151^

In 1963, Robert F. Shaw, a surgeon and inventor at San Francisco Presbyterian Hospital (San Francisco, California, United States), constructed the first absolute reading eight-wavelength ear oximeter and resolved the calibration issue. The multi-wavelength device was capable of determining the relative amounts of the Hb derivatives oxy-, deoxy-, carboxy-, and methemoglobin. The light absorption at each wavelength permitted the concentrations to be calculated using a simultaneous set of Beer’s Law equations. The expansion of the wavelength range led to enhanced accuracy. In November 1967, Show filed a patent application, which was subsequently granted in 1972.^152^ In 1974, following the initial success of Shaw’s oximeter, Hewlett-Packard (Palo Alto, California, United States) constructed the first commercial eight-wavelength (650 to 1050 nm) fiber-optic ear probe oximeter (Model 47201A)^145^ [Fig. 6(b), 6(c)]. The arterialization of the ear was achieved by heating the skin to a temperature of 41°C. No calibration was required, and the accuracy was 1.7% above 90% ${\mathrm{SaO}}_{2}$. Eight wavelengths were sufficient to differentiate all forms of Hb, including sulfhemoglobin. The Hewlett-Packard oximeter was expensive ($13,000), cumbersome, heavy, and large and required a significant operator input to operate. The device was primarily employed in sleep laboratories, in specialist pulmonary laboratories, and to a considerable extent in the acceleration training of aviators and astronauts.^153^ The oximeter was relatively insensitive to changes in earpiece position and differences in skin pigmentation,^154^ but had the problem of providing too low readings when $O_{2}$ saturation was <65%.^155^ Bilirubin was also found to interfere with the measurements,^156^ and compared to transcutaneous oximetry in a study in neonates, it was found to be “more difficult to maintain contact between sensor and subjects for long periods.”^157^ The Model 47201A later became the “gold standard” for comparison with other oximeters, but due to its large earpiece and high cost, it was hardly used for clinical monitoring. The instrument was produced until 1983.

In 1970, the American Optical Company (AO) (Southbridge, Massachusetts, United States) introduced two reflectance oximeters, the AO Macro Oximeter II and the AO Micro Oximeter II, which were capable of determining ${\mathrm{SaO}}_{2}$ from up to 0.2 ml of a blood sample in ∼20  s. These AO oximeters were used as point-of-care tests at the patient’s bedside. In 1972, Arnon Cohen, an electrical engineer (University of Connecticut, Storrs, Connecticut, United States), developed a two-wavelength instrument for monitoring relative blood $O_{2}$ saturation in the skin using backscattered light^146^ [Fig. 6(d)]. The instrument comprised a miniature optical transducer, equipped with red and near-infrared LEDs and a photodetector. This was the first oximeter to utilize LEDs as a light source. The first practical visible spectrum LED was invented in 1962 by Nick Holonyak, Jr. (1928–2022) at the General Electric electronics laboratory in Syracuse (New York, United States). In 1973, Dietrich Werner Lübbers (1917–2005) at the Max Planck Institute for Systemphysiologie (Dortmund, Germany) developed a complex mathematical model for reflection oximetry from living tissue, which overcame the problem of skin pigmentation.^158^^,^^159^ It is noteworthy that Lübbers’s scientific career commenced in 1941 with the research project for his medical dissertation at the Institute of Physiology at the University of Berlin (Berlin, Germany), under the supervision of Kramer.

### 3.4 1972–1988: Aoyagi’s Pulse Oximetry Discovery and the First Commercial Devices

**Fig. 7:**
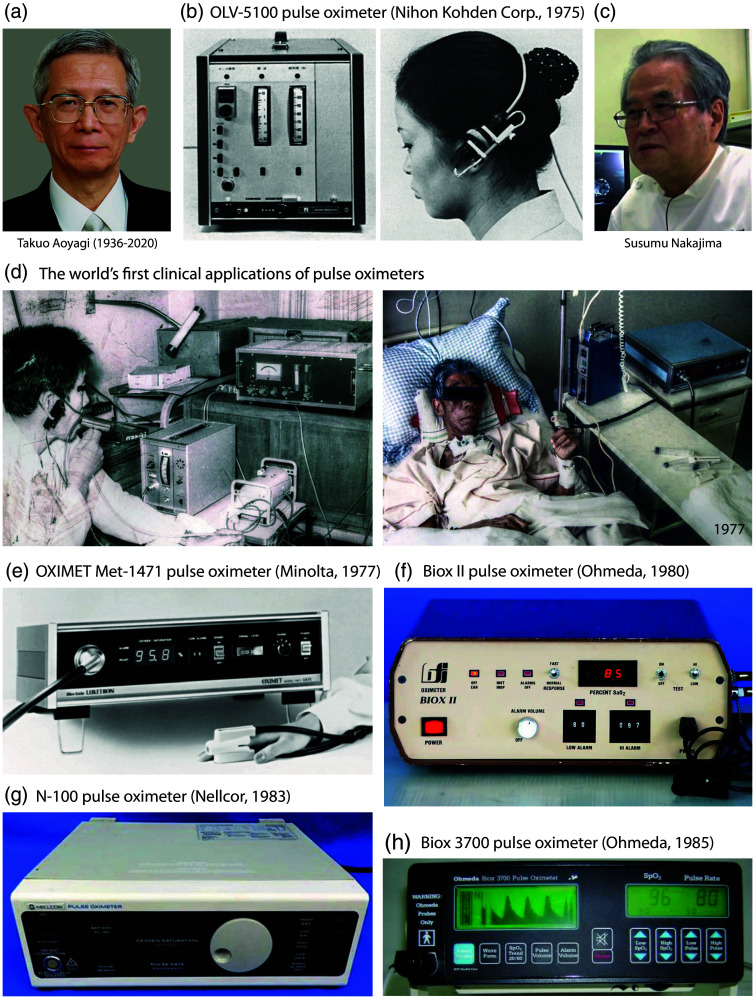
Examples of POs developed in the 1970s and 1980s. (a) Picture of Takuo Aoyagi. Reprinted with permission from IEEE. (b) OLV-5100 PO by Nihon Kohden Corporation, patented by T. Aoyagi. Shown is the main part of the device (left) and the ear probe attached (right). Reprinted from Severinghaus and Honda,^6^ with permission from the publisher. (c) Picture of Susumu Nakajima. Reprinted with permission from NHK World-Japan. (d) The world’s first clinical applications of POs: the test of Aoyagi’s device by S. Nakajima at Sapporo Misumai National Sanatorium with a patient with respiratory failure after thoracoplasty for tuberculosis treatment (left) and the first clinical application of a finger-clip PO (OXIMET Met-1471) at Misumai Sanatorium by S. Nakajima in a patient suffering from hypoxemia post-surgery. Both images were obtained by S. Nakajima, with permission for publication. Both original photos have been edited by us to improve the quality (color and contrast). (e) OXIMET Met-1471 PO (Minolta Camera, Co., Ltd.). Reprinted from Miyasaka et al.,^46^ with permission from the publisher. (f) Biox II PO (Ohmeda). Picture from pulmonary function tests history.^160^ (g) N-100 PO (Nellcor). Reprinted from Miyasaka et al.,^46^ with permission from the publisher. (h) Biox 3700 PO (Ohmeda). Picture from DOTmed WebStore.^161^

PO, as it is currently understood, was first conceptualized in December 1972 by Takuo Aoyagi (1936–2020) [Fig. 7(a)], an electrical engineer at Nihon Kohden Corporation (Tokyo, Japan). Nihon Kohden was established on August 7, 1951, by Yoshio Ogino to integrate the expertise of electrical engineering with that of medicine. Ogino studied medicine and commenced work in the field of medical electronics around 1945. From 1959 to 1971, Aoyagi was employed by Shimadzu Corporation in Kyoto. In 1971, he joined the R&D department of Nihon Kohden as head of a group tasked with developing new biomedical technologies, with a particular focus on a non-invasive dye densitometer for cardiac output measurement. In December 1972, 30 years after the publication of Glenn Millikan’s seminal article in 1942 describing the first portable ear oximeter, Aoyagi, while working on a device, observed pulsatile artifacts in the absorbance of intravenous indocyanine green dye, which was recorded using light transmission through the ear of a Japanese version (Erma) of Wood’s ear oximeter.^162^^–^^166^ This is the fundamental principle underlying Aoyagi’s serendipitous PO invention. It seems probable that Aoyagi was unaware of the observations published 15 years earlier by the American anesthesiologist Seymour Schotz and colleagues, which identified a wave-like pattern associated with pulsatile blood flow to the ear in the near-infrared recording of the Wood ear oximeter.^142^^–^^144^ Nevertheless, it was the seminal work of Aoyagi that finally made non-invasive optical oximetry a viable clinical option. He discerned that these pulsatile alterations could be employed to calculate ${\mathrm{SaO}}_{2}$ using the ratio of pulse changes in the red and infrared spectral regions. Aoyagi was aware of the significance of this and proceeded to develop a dual-wavelength ear PO, which utilized the pulsating blood flow to detect and measure arterial blood absorption for the determination of ${\mathrm{SaO}}_{2}$.

On April 26, 1974, Aoyagi delivered an oral presentation on the development of the ear PO at a conference of the Japan Society of Medical Electronics and Biological Engineering (MEBE) in Osaka. The abstract was submitted in October 1973 and accepted in January 1974.^164^ In a prudent move, Takuo Aoyagi and Michio Kishi submitted a Japanese patent application entitled “Optical Type Blood Measuring Equipment” on March 29, 1974 (patent number JPS5326437) prior to the presentation at the Osaka meeting. The patent was duly granted in 1979. Unfortunately, the patent was not extended to other countries. The PO did not necessitate a bloodless calibration and provided reproducible ${\mathrm{SaO}}_{2}$ readings.

In early 1973, Susumu Nakajima [Fig. 7(c)], a young surgeon working at the Sapporo Misumai National Sanatorium, became acquainted with Aoyagi’s idea through a conversation with Mr. Y. Sugiyama, Director of Development at Nihon Kohden Corporation. With a budget of 2.5 million yen, Nakajima requested that the company manufacture a custom-designed ventilator for clinical testing as part of the establishment of an intensive care unit. On March 28, 1973, Nakajima received a letter from Aoyagi in which the latter set forth his initial ideas, which were conceived a year prior to the filing of the patent. The PO prototype was produced between September 1973 and March 1974. In the spring of 1974, Nakajima and his colleagues Yasuro Hirai, Hiroshi Takase, and Akihiko Kuze conducted preliminary tests on the Nihon Kohden Corporation ear PO prototype N.1 on a dog and subsequently on a patient undergoing thoracoplasty for tuberculosis treatment [Fig. 7(d)]. The concentration of blood $O_{2}$ was continuously monitored during the administration of $O_{2}$ via the PO, while ${\mathrm{pCO}}_{2}$ was measured continuously with a capnometer. In addition, other measurements were conducted during light exercise and following the administration of acetazolamide (a carbonic anhydrase inhibitor). These constituted the first clinical applications of PO. The results were presented at a conference in Tokyo in 1975^167^^–^^169^ and published in the same year, with the Nihon Kohden developers Takuo Aoyagi, Micho Kishi, and Kazuo Yamaguchi as co-authors.^170^ This invention led to the rapid development of PO; however, Aoyagi did not continue to work on the invention as he had to leave his research group for 8 years. At the end of Aoyagi’s scientific career, on June 29, 2015, he was awarded the honorary IEEE Annual Medal for Innovations in Healthcare Technology at the IEEE Honors Ceremony at the Waldorf Astoria hotel in New York, United States.

In 1973, Minolta Camera Company Ltd. (Osaka, Japan) (now Konica Minolta, Inc.) initiated research into oximetry. In 1973, Morimasa Takeda reported that the pulse wave obtained by photoelectric measurement of red light transmitted through the fingertip represented the fluctuation of blood thickness according to pulsation.^171^ The measurement was conducted using a photoelectric pulse wave meter (photoplethysmograph), which employed an LED and a silicon photodiode. This instrument served as the precursor to the OXIMET Met-1417 PO, which was released in 1977. In 1974, the Minolta team became aware of the possibility of developing a non-invasive device for measuring ${\mathrm{SaO}}_{2}$ and filed a patent for Japan in 1974 and for the United States in 1975,^172^ having independently conceived the idea without any input from Nihon Kohden Corporation, Tokyo, Japan. In 1979, a USA patent was filed by Minolta for a novel designed digital PO.^173^

In 1975, Nihon Kohden Corporation launched the first dual-wavelength ear PO, the OLV-5100 [Fig. 7(b)], which was patented by Takua Aoyagi.^46^^,^^174^ The transmission of light signals from the device to the ear was achieved via heavy fiber optic cables, which presented a challenge in maintaining the position of the ear. The accuracy of the device was limited by the simplified calibration process. The device was not a commercial success due to its weight and fragility.

In 1977, Minolta Camera Company Ltd. introduced the first dual-wavelength fingertip PO model, the OXIMET Met-1471. In June, the PO (equipped with a tungsten lamp, silicon photodiode, and fiber optic cables) was launched in Japan through Mochida Pharmaceuticals, Tokyo, Japan. Unfortunately, the probe and the fiber optic cables were found to be unsuitable for use in clinical monitoring due to their size and weight.

In 1977, Nakajima et al. tested OXIMET Met-1471 on a patient who had undergone postoperative radiotherapy for esophageal cancer [Fig. 7(d)].^175^ At the time, he was located at the Asahikawa Medical University Hospital in Asahikawa City, a town situated 140 km north of Sapporo. Frank Sarnquist validated the Minolta PO 101 device (identical to the OXIMET Met-1471) at Stanford University (Stanford, California, United States) and demonstrated that the ${\mathrm{SpO}}_{2}$ determined correlated well with $O_{2}$ saturation by *in vitro* oximetry for ${\mathrm{SaO}}_{2}>90\%$.^176^ In Japan, however, the PO was considered a useful research device but not a clinically viable option; only ∼200 OXIMET Met-1471 devices were sold. Nevertheless, the initial PO prompted the market to enhance oximetry technology.

Since 1979, the development of PO devices has taken place in California (United States). In 1979, engineer Scott A. Wilber co-founded Biox Technology Inc. (Boulder, Colorado, United States) and developed the first commercial PO, which used LEDs as light sources and photodiodes as detectors in an ear clip. The US patent was filled in 1981.^177^ Wilber replaced the Beer-Lambert law with a calibration curve that permitted the estimation of ${\mathrm{SaO}}_{2}$ to be made with greater accuracy across the entire range. The incorporation of a microprocessor enhanced the empirical calibration and accuracy. The patented normalization circuit proved to be a significant and long-lasting innovation in PO. The Biox II [Fig. 7(f)], a PO with earlobe measurement,^178^ introduced in 1980, was initially used by respiratory care and later by anesthetists. Subsequent PO models were Biox IIa and Biox III.

In 1980, I. Yoshiya and colleagues from the Osaka University Hospital (Osaka, Japan) published the development of a fingertip PO with a good linearity, a measurement error within ±5%, but with the disadvantage that it was susceptible to finger movements in relation to the finger sensor.^179^

In 1980, Setsuo Takatani and Peter Cheung (Cleveland, Ohio, United States) published the development of a five-wavelength reflectance oximeter that was capable of measuring both ${\mathrm{SaO}}_{2}$ and total Hb.^180^

In 1981, Nellcor Inc. (Hayward, California, United States) introduced the first finger PO device for routine use in the operating room. The company was established by William New (1942–2017), Jack Lloyd Jim, Jim Corenman, and Bob Smith. New was a Stanford University anesthesiologist who played a pivotal role in the promotion of the Biox II PO during anesthesia. New recognized the potential of the device beyond its role in respiratory care and thus founded the company with the objective of improving the PO and promoting its use in the operating room. In 1982, Nellcor Inc. produced the prototype N-100A, which featured a small finger probe and employed LEDs and a silicon photodiode as a detector [Fig. 7(g)]. The PO model was strikingly similar to Minolta’s OXIMET Met-1471 fingertip PO device. In 1982, Nellcor commenced the manufacture and sale of the N-100 PO device for surgical use on a global scale. The PO device included an audible indicator that provided information on both the pulse rate and ${\mathrm{SpO}}_{2}$. As the ${\mathrm{SpO}}_{2}$ value fluctuated, so did the pitch of the pulsing sound. The Nellcor N-100 was subjected to several studies for evaluation.^181^^–^^187^

Nellcor rapidly gained market share from Biox. The N-180, which was launched in 1987, incorporated a novel feature whereby the readings were updated with each pulse beat. The company subsequently launched a series of other POs (N-180, N-20, N-200, N-3000). In July 1997, Nellcor was acquired by Mallinckrodt Inc. In 2000, Mallinckrodt was acquired by Tyco International Ltd. In 2007, Covidien became a public company and continued to market the Nellcor brand, which, along with Masimo (Irvine, California, United States), dominated the market. In 2015, Medtronic acquired Covidien and continued to market the Nellcor brand. The company currently offers several PO models on the market, including a portable PO (PM10N) and a PO for bedside patient monitoring (PM100N).

In the summer of 1984, the British Oxygen Company purchased Biox Technology, which subsequently became a division of the British Oxygen Company’s medical group, Ohmeda (Boulder, Colorado, United States). Wilber, the technical founder of Biox Technology, ceased his involvement with the company. The Biox III product was subsequently renamed the Ohmeda Biox 3700, representing a significant advancement in portable, battery-operated PO technology. The Ohmeda Biox 3700 was the first such device to display both numerical and graphical representations of ${\mathrm{SaO}}_{2}$ and pulse rate at a given moment, as well as changes in ${\mathrm{SaO}}_{2}$ over the past hour [Fig. 7(h)]. The device was equipped with ear and finger sensors for use with adult patients, while smaller sensors were employed for children and infants. In addition, the device was equipped with both visual and audible alarms, as well as a backup battery. By the mid-1980s, the device had become a widely used anesthesia market product. A number of studies were conducted to evaluate the device.^181^^,^^186^^–^^194^

In 1984, Minolta’s OXIMET Met-1471 PO camera was calibrated using a model cell into which blood or Hb solution was pumped in and out using a rotary pump.^195^ The device was found to be effective in compensating for the effect of multiple scattering, thereby significantly enhancing the accuracy of PO.

In May 1985, a 3-day symposium on the clinical applications of oximetry was held at Chartridge (Buckinghamshire, United Kingdom), organized by the Research Department of Anaesthetics of the Royal College of Surgeons of England and Ohmeda.^8^

In 1986, PO became a standard component of patient monitoring during anesthesia, initially at Harvard Medical School (Cambridge, Massachusetts, United States) and subsequently throughout the United States.^196^ The utilization of PO was rapidly disseminated to additional hospital departments, including emergency rooms, recovery rooms, neonatal units, and intensive care units. The advent of PO was accompanied by a 90% decline in anesthesia-related fatalities. In 1988, ${\mathrm{SpO}}_{2}$ measured with PO was proposed to be considered as a “fifth vital sign” along with temperature, blood pressure, pulse, and respiratory rate.^197^ In 1988, an international ISO standard on PO (IES 60601-1:1988) for the safety and performance of PO equipment for medical use came into force. In 2005, the previous standard, ISO 60601-1:1988, was superseded by ISO 9919:2005, which sets out the specific safety and performance requirements for PO equipment used in medical settings.

Also in 1986, physicist Phil Isaacson and three engineers established Nonin Medical Inc. (Plymouth, Minnesota, United States). The first PO model was the 8604. In the late 1980s, Nihon Kohden Corporation resumed research and development of PO. The PO model PO OLV-1100 was launched in 1988, followed by the model OLV-1200.

A detailed historical review of oximetry was published in 1986 by John W. Severinghaus and Poul Bjørndahl Astrup (1915–2000) (University of Copenhagen, Copenhagen, Denmark), the founders of modern blood gas analysis.^12^ Severinghaus is credited with the invention of the triple-function blood gas analyzer.^5^^,^^12^ In 1957, at the National Institutes of Health (NIH) in Bethesda (Maryland, United States), Severinghaus and the technician Freeman Bradley, Jr. combined the ${\mathrm{CO}}_{2}$ electrode of Richard W. Stow (1916–1995) (Ohio State University Hospital, Columbus, Ohio, United States) with the $O_{2}$ electrode of Leland C. Clark, Jr. (1918–2005) (Children’s Hospital Medical Center, Cincinnati, Ohio, United States) to create the first blood gas analyzer. In 1959, the addition of a pH electrode enabled the measurement of acidity or alkalinity in a blood sample. In 1987, Wukitsch published a further historical review of PO, in which he summarized the developments made by the Ohmeda company.^59^

In 1988, Nihon Code Corporation introduced the PO model PO OLV-1100, which was subsequently replicated by the model OLV-1200.^46^ In the same year, Ohmeda introduced the 4700 OxiCap monitor, which combined a PO, a capnograph (measuring ${\mathrm{CO}}_{2}$ in expired air, i.e., end-tidal ${\mathrm{CO}}_{2}$, $P_{\mathrm{ET}}{\mathrm{CO}}_{2}$) and an inspired $O_{2}$ concentration (${\mathrm{FiO}}_{2}$) monitor in one portable unit. Measurements were displayed on the screen in the form of graphs and numerical values. In 2003, GE HealthCare acquired Instrumentarium (including its Datex-Ohmeda division).

The first detailed reviews of the clinical applications of PO and the limitations of its performance were published in 1987 by Steven J. Barker and Kevin K. Tremper (Irvine Medical Center, University of California, California, United States)^49^ and in 1989 by Joseph F. Kelleher (Scripps Mercy Hospital, San Diego, California, United States).^37^ In the same year, the first examples of the Ohmeda Biox 3700 ambulatory PO, powered by battery, for unsupervised long-term monitoring of ${\mathrm{SaO}}_{2}$ were published.^198^^,^^199^ In the 1980s, numerous studies were published investigating the accuracy and reliability of commercially available POs.^181^^,^^184^^,^^198^^–^^210^

### 3.5 1989–2019: Thirty Years of Pulse Oximetry Development

**Fig. 8:**
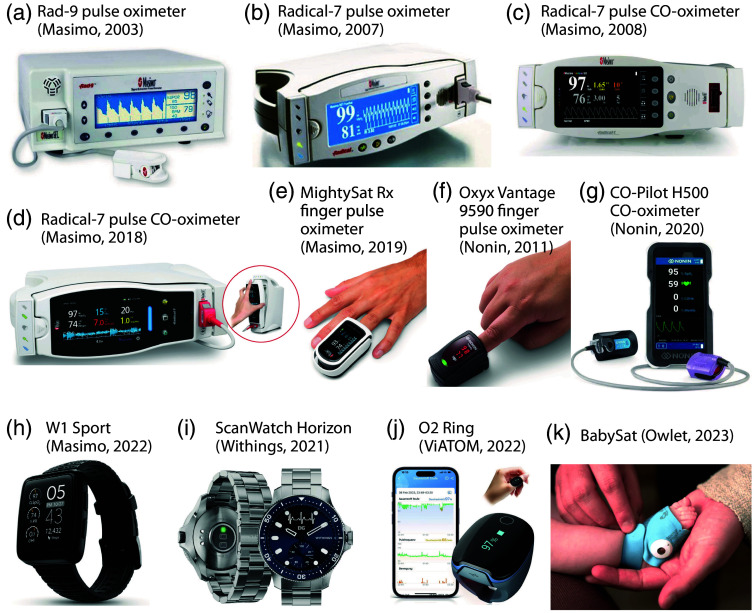
Examples of POs and pulse CO-oximeters developed in the 2000s, 2010s, and 2020s. (a) Rad-9 PO (Masimo). (b) Radical-7 PO. (c), (d) Two versions of the Radical-7 pulse CO-oximeter with the rainbow SET technology. (e) MighySatRx finger PO (Masimo). Images (a)–(e) from the Masimo website. (f) Oxyx Vantage 9590 finger PO (Nonin). (g) CO-Pilot H500 pulse CO-oximeter (Nonin). Images (f) and (g) from the Nonin website. (h) W1 Sport PO smart watch (Masimo). Image from the Masimo website. (i) Scan Watch Horizon PO smart watch (Withings). Image from the Withings website. (J) $O_{2}$ Ring (ViATOM). Image from the ViATOM website. (K) BabySat (Owlet). Image from the Owlet website.

Masimo, a start-up company, was founded in 1989 by electrical engineer Joe Kiani (1964–). Kiani and Mohamed Diab invented the modern PO by developing a novel PO measurement approach and signal processing algorithm, the Massimo Signal Extraction Technology (SET).^211^ The method does not rely on the assumption made by conventional PO that arterial blood is the sole pulsatile component that absorbs light in the tissue under investigation. The discrete saturation transform is employed to isolate individual “saturation components” within the optical path, thereby enabling the cancellation of varying absorption changes that are not due to arterial pulsation. Such changes may be induced, for instance, by patient motion. Furthermore, the method enhanced the precision of PO measurement during exercise and low perfusion. In 1996, the first model employing the SET was released. In 1998, Masimo received FDA 510(k) clearance for SET measure-through-motion PO technology, which enabled PO measurement during phases of motion. In 1999, Masimo obtained FDA 510(k) clearance for SET low-perfusion technology, which improved PO measurement under conditions of low tissue perfusion. A study from 2002 demonstrated that the SET method significantly improves PO measurement during the motion of the subject.^212^ In 2005, Masimo launched the first multi-wavelength pulse CO-oximeter (Rainbow SET), which was capable of measuring ${\mathrm{SpO}}_{2}$, pulse rate, respiratory rate, respiratory effort, total Hb, COHb, MetHb, perfusion index, and $O_{2}$ reserve index. In 2006, the FDA granted clearance for the Radical-7 pulse CO-oximeter with Rainbow technology. Figures 8(a)–8(e) illustrate a selection of Masimo PO and pulse CO-oximetry devices.

In 1990, Nonin Medical Inc. launched the first portable handheld PO (Model 8500). In 1992, the same company constructed the inaugural MRI-compatible fiber-optic PO, comprising non-magnetic components to monitor patients during MRI scans. In 1995, Nonin introduced the first fingertip PO, comprising all components in a single unit that could operate for up to 18 h. In 2008, Nonin Medical Inc. introduced the Onyx II (model 9560), the first fingertip PO with Bluetooth wireless technology. In 2011, the Onyx Vantage 9590 professional fingertip PO was launched, which also functions in low perfusion. In 2013, Nonin Medical Inc. released the first universal oximetry system combining PO and regional cerebral oximetry (based on near-infrared spectroscopy, NIRS) in one system with Bluetooth low-energy wireless technology (Nonin CO-Pilot H500 and Nonin 3500 SP). The CO-Pilot H500 handheld multiparameter pulse CO-oximeter system simultaneously measures ${\mathrm{SpO}}_{2}$, pulse rate, COHb, and MetHb [Fig. 8(g)]. Nonin also developed a novel finger PO [Fig. 8(f)].

Triton Electronic Systems Ltd. (Ekaterinburg, Russian Federation) was established in 1989 by researchers from the Ural Scientific School. The company’s first PO (OP-31) was launched in 1992 in collaboration with anesthesiologist Boris Zislin.^137^

On September 7, 1992, the FDA issued a general guidance document on noninvasive POs: “POs – Premarket Notification Submissions [510(k)s] Guidance for Industry and FDA Staff,” last updated in March 2013. In 2017, ISO 80601-2-61:2017 “Medical electrical equipment - Part 2-61: Particular requirements for basic safety and essential performance of PO equipment” was published.

By 1993, 35 PO manufacturers were listed, the cost of POs had fallen to less than $1500 and PO’s accuracy was ∼2% for ${\mathrm{SaO}}_{2}>70\%$.^7^ Over the course of these decades, hundreds of new PO companies were established, yet only a select few have made a notable contribution to the advancement of the technology. The most recent generation of POs is capable of providing accurate measurements in challenging circumstances, such as low perfusion, the presence of motion artifacts, and low ${\mathrm{SaO}}_{2}$. Recent advances have focused on the analysis of the morphological characteristics of the PPG waveform, which is used to identify new parameters that may have clinical significance. These include the PPG variability index and the perfusion index.

In 2000, the US Centers for Medicare and Medicaid Services accepted physician billing for in-office PO measurements. In 2007, the World Health Organization (WHO) (Geneva, Switzerland) included PO as an essential component of its surgical safety checklist to reduce complications.

In 2003, it was demonstrated that it is possible to determine the respiratory rate from the PPG signal produced by a standard PO (in this case a Nellcor N-100) using the wavelet transform.^213^ A year later, the researchers were able to confirm that their technique was indeed capable of identifying each breath in the PO signal.^214^

In 2007, Humphreys et al.^215^ published the development of a camera-based non-contact dual-wavelength PPG device, which could be used as a non-contact PO.

In 2014, Huang et al.^216^ reported on the development of a wearable and wireless ring-type PO with a multi-detector.

In 2018, Zhao et al.^217^ published the invention of a high-speed (up to 27 Hz) frequency-domain spectroscopy technique with five wavelengths and deep neural network signal processing for the quantitative determination of $O_{2}\mathrm{Hb}$ and HHb. In the same year, Hay et al.^218^ reported the construction of a modified PO with two nearby infrared LEDs with wavelengths that have relatively similar optical scattering constants and path lengths in the tissue, thus not requiring calibration.

In 2019, Tamura^219^ published a review of the developments and clinical practice of wearable PPG and PO devices, as well as various methods of eliminating motion artifacts. In the same year, Von Chong et al.^220^ reported the development of a PO with only a single LED and a buried quad junction photodetector, i.e., a multispectral sensor.

In 2015, the Owlet Smart Sock wireless PO device (Owlet, Inc., Lehi, Utah, United States) was introduced into the market, enabling the measurement of ${\mathrm{SpO}}_{2}$ and HR in neonates. The product was well received by parents and found to be useful in monitoring the health state of the infant and improving the sleep quality of the parents.^221^

### 3.6 2020–2024: Pulse Oximetry During the Pandemic and the Need for Further Improvements in Pulse Oximetry Technology

Since 2020, a number of new PO innovations have been introduced. Some smartwatches are now able to measure ${\mathrm{SpO}}_{2}$ on the wrist or finger. For example, in 2020, Apple, Inc. (Cupertino, California, United States) incorporated ${\mathrm{SpO}}_{2}$ measurement functionality into its smartwatches, specifically the Series 6. In that year, Masimo initiated legal proceedings against Apple, alleging patent infringement. In 2023, Apple commenced the sale of versions of the Series 9 and Ultra 2 watches in the United States, which lacked PO functionality due to ongoing legal disputes with Masimo. In 2021, the French company Withings (Issy-les-Moulineaux, France), founded in 2008 by the French engineer Éric Carreel (1959-), introduced the first FDA-approved PO wristwatch, the ScanWatch, and the ScanWatch Horizon [Fig. 8(i)].

Figures 8(a)–8(d) show the development of modern PO and pulse CO-oximeters from Masimo. In 2019, Masimo announced FDA clearance of the measurement of respiratory rate on the new MightySat Rx fingertip PO [Fig. 8(e)], in 2022, the smart watch W1 Sport with PO functionality [Fig. 8(h)], and in 2022, the company ViATOM released a PO in the form of a ring [Fig. 8(j)].

From 2020 onward, the COVID-19 pandemic brought a whole new dynamic to the development and application of PO and pulse CO-oximetry.^3^ The application of PO in relation to COVID-19 soon became evident as a valuable diagnostic and monitoring tool, as evidenced by several publications published already in 2020.^54^^,^^222^^–^^228^

In the same year, we published a letter highlighting the potential for the use of PO in the detection of COVID-19 pneumonia associated with “silent hypoxia.”^229^ Furthermore, in the current year, it was discovered that a subset of patients diagnosed with COVID-19 exhibited a normal respiratory rate (RR), despite significant hypoxia. This observation has led to the hypothesis that the ${\mathrm{SpO}}_{2}$/RR quotient might be a particularly useful metric for evaluating “silent hypoxia” in the context of the disease.^230^ In addition, the discrepancy between ${\mathrm{SpO}}_{2}$ and ${\mathrm{SaO}}_{2}$ readings has been demonstrated to possess diagnostic value in the assessment of microvascular thrombosis in patients with COVID-19, as highlighted by Satici et al.^231^

In 2020, we also proposed that cerebral NIRS-based oximetry should be explored as a warning indicator for mechanically ventilated adults with COVID-19.^232^ Moreover, in 2023, we emphasised that the potential influence of skin pigmentation on cerebral NIRS-based oximetry should also be taken into account.^233^

In 2020, researchers also emphasized that when utilizing PO in the context of the COVID-19 pandemic, it is essential to consider that there are still challenges and potential for errors when employing PO.^222^^,^^234^ The accuracy of PO measurements can be influenced by a number of factors, including skin perfusion, skin temperature, skin thickness, anemia, the presence of dysfunctional Hb, and skin pigmentation.^222^^,^^235^

Concerning skin perfusion, according to a recent systematic review, POs are generally “accurate in poorly perfused patients, especially newer oximeter models and those placed on earlobes.”^236^ Skin temperature is another factor affecting accuracy since cold skin temperature is associated with reduced blood volume and flow, which in turn reduces the quality of the measurement of the blood volume pulsation amplitude difference between the red and IR absorption signals and thus the determination of ${\mathrm{SpO}}_{2}$. According to experimental findings reported by Kan et al.,^237^ a warm skin temperature (about 33°C) should be maintained for accurate PO measurements. A recent systematic review of the literature on the impact of nail polish on the accuracy of PO found that nail polish can affect the determination of ${\mathrm{SpO}}_{2}$. However, the effect was categorized as being “clinically insignificant.”^238^

**Fig. 9:**
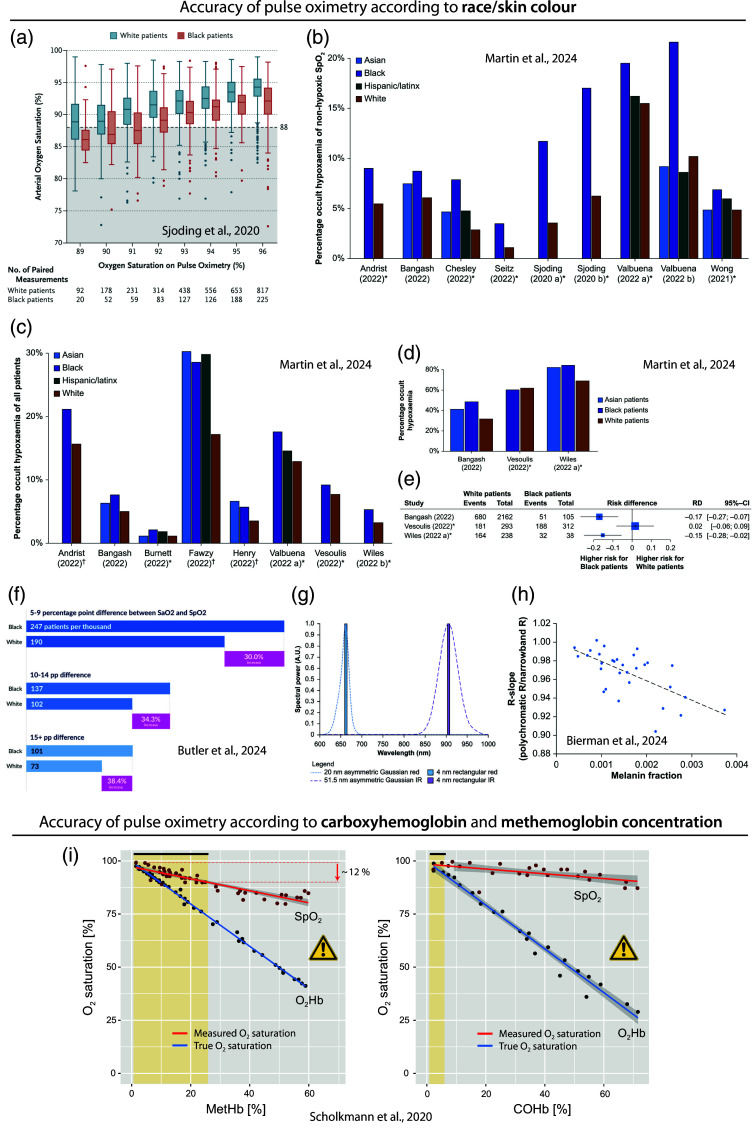
Problems with the accuracy of PO. (a) Bias in ${\mathrm{SpO}}_{2}$ readings in Black patients compared to White patients according to the study of Sjoding et al.^45^ The ${\mathrm{SpO}}_{2}$ values are plotted in comparison to the ${\mathrm{SaO}}_{2}$ values measured in parallel. Ideally, both values should be identical. A larger discrepancy can be seen in the Black patients compared to White patients. Reprinted from Sjoding et al.,^45^ with permission from the publisher. (b)–(e) Results of the systematic review of Martin et al.^239^ (b) Frequency (in %) of occult hypoxemia in paired ${\mathrm{SpO}}_{2}–{\mathrm{SaO}}_{2}$ measurements among readings/patients with non-hypoxemic ${\mathrm{SpO}}_{2}$. (c) Frequency (in %) of occult hypoxemia in paired ${\mathrm{SpO}}_{2}–{\mathrm{SaO}}_{2}$ measurements out of all patients. (d) Clinical studies with respective percentages of readings with occult hypoxemia out of readings/patients with true hypoxemia. In panels (b)–(e), it can be seen that Blacks have generally a higher value, indicating a PO bias. (e) Forest plot visualizing the risk difference for Black and White patients of occult hypoxemia compared to true hypoxemia. Panels (b)–(e) reprinted from Martin et al.,^239^ with permission from the publisher. (f) Study results of Butler et al.^240^ Reprinted from Butler et al.,^240^ with permission from the publisher. (g), (h) Results of the simulation study of Bierman et al. showing that the spectral bandwidth (visualized in panel (g) as either Gaussian functions of LED or discrete values of laser diodes) affect how strong a PO is biased by melanin present in the tissue (h). Reprinted from Bierman et al.,^241^ with permission from the publisher. (i) Effect of the MetHb and COHb concentration in the blood on the accuracy of ${\mathrm{SpO}}_{2}$ determined with a PO; reprinted from Scholkmann et al.,^242^ with permission from the publisher. MetHb: methemoglobin, COHb: carboxyhemoglobin, $O_{2}\mathrm{Hb}$: oxyhemoglobin, ${\mathrm{SaO}}_{2}$: arterial hemoglobin $O_{2}$ saturation, ${\mathrm{SpO}}_{2}$: peripheral arterial hemoglobin $O_{2}$ saturation.

Moreover, during the pandemic, there was a growing recognition and investigation of the fact that the accuracy of PO can depend on the race and ethnicity of the subject being measured. This issue was brought to the fore by a study published by Sjoding et al.^45^ in the *New England Journal of Medicine* with the title “Racial bias in pulse oximetry.” In this study, patients who identified their race as Black or White were tested for occult hypoxemia (i.e., ${\mathrm{SaO}}_{2}$ of <88% despite ${\mathrm{SpO}}_{2}$ of 92% to 96% measured with PO). The study revealed that Black patients exhibited a nearly threefold higher prevalence of occult hypoxemia than White patients, suggesting the potential for racial bias in PO [Fig. 9(a)]. In a recent systematic review of studies up to March 2023, Martin et al.^239^ analyzed 44 studies and concluded that the data support the notion that PO “can overestimate true ${\mathrm{SaO}}_{2}$ in people with darker skin tones” [Figs. 9(b)–9(e)].

The influence of race, ethnicity, and skin pigmentation on PO measurements has been a topic of discussion for several decades. For example, in 1988, Cecil et al.^181^ conducted a study comparing the accuracy of two pulse oximeters (the Nellcor N-100 and the Ohmeda 3700) with that of CO-oximetry. The findings demonstrated that the measurements exhibited reduced precision in Black people compared to White people.

Subsequently, in 1989, Riess et al.^243^ demonstrated that ear POs exhibited reduced accuracy and a higher incidence of technical issues in individuals with darker skin tones compared to those with lighter skin tones. However, these findings did not prompt the manufacturers of POs to take the issue seriously and implement measures to optimize the technology to eliminate the bias. In accordance with the current FDA recommendations for premarket clinical studies, only 15% of the subjects used to validate and calibrate the PO device need to be of darker pigmentation.^244^

In 1990, Jubran and Tobin^245^ demonstrated that the optimal ${\mathrm{SpO}}_{2}$ target value to avoid hypoxemia in ventilator-dependent patients was dependent on the patient’s skin color. For White patients, the optimal ${\mathrm{SpO}}_{2}$ target value was 92% ${\mathrm{SpO}}_{2}$, while for Black patients, it was 95% ${\mathrm{SpO}}_{2}$. Furthermore, it was found that inaccurate PO readings (i.e., ${\mathrm{SpO}}_{2}–{\mathrm{SaO}}_{2}>4$ percent points) were found to be more common in Black patients than in White patients.

A recent study on this type of bias in POs, based on a large dataset of 13,483 patients, has confirmed the problem. The study found that non-Hispanic Black patients were 32% more likely to have occult hypoxemia than White patients, and that these results “support the need for further investigation of pulse oximeter accuracy to account for differences in patient skin pigmentation, race, and ethnicity as suggested by the FDA”^240^ [Fig. 9(f)]. Another recently published study demonstrated that individuals with darkly pigmented skin exhibited a greater PO bias compared to those with lighter pigmentation. Furthermore, the study revealed that the PO error (${\mathrm{SpO}}_{2}–{\mathrm{SaO}}_{2}$) for individuals with darkly pigmented skin increased as ${\mathrm{SaO}}_{2}$ decreased.^246^

The biophysical reason for the impact of skin pigmentation on the accuracy of POs is intricate. As has recently been demonstrated, the spectral bandwidth of the light sources used is likely to be a significant factor^241^ [Figs. 9(g), 9(h)]. It can be seen that ensuring a more diverse population, in terms of race and ethnicity, and skin pigmentation, in the empirical calibration of POs will only serve to partially address the issue of bias.

In recent times, a number of governmental agencies and the US FDA have issued guidelines to address the issue of bias in POs. Nevertheless, several authors have indicated that skin pigmentation continues to influence the accuracy of POs on the market. Following the publication of these data, the FDA Safety Communication in February 2021 informed patients and healthcare professionals that POs have limitations and may provide inaccurate results in certain circumstances, including due to skin pigmentation.^247^ In November 2022, the FDA convened a virtual public meeting of the CDRH Anesthesiology and Respiratory Therapy Devices Panel of the Medical Devices Advisory Committee. The purpose of the meeting was to share information and perspectives from interested parties about ongoing concerns that POs may be less accurate in individuals with darker skin pigmentation.^248^ At a subsequent meeting of the FDA in February 2024, “the panel agreed with DFA’s [Doctors for America’s] proposed approach to improve the quality of premarket studies and associated methods used to evaluate the performance of pulse oximeters taking into consideration a patient’s skin pigmentation, race, and ethnicity.”^249^ Recently, FDA researchers also presented the use of melanometry to objectively assess skin pigmentation in pulse oximetry studies.^250^

Another issue with PO that has become more pertinent in the context of the COVID-19 pandemic is the potential impact of dysfunctional Hb (i.e., MetHb and COHb) on the accuracy of PO. Given that acute COVID-19 disease can result in elevated levels of MetHb and COHb in the blood, this can negatively impact the precision of PO ${\mathrm{SpO}}_{2}$ measurements, as previously emphasised by us in a related publication^242^ [Fig. 9(i)]. One solution to this problem is to use pulse CO-oximeters instead of classic POs.

In a recent review of new studies published in 2022 and 2023 about bias and precision of PO, Dean R. Hess^63^ concluded that bias and precision are issues with PO. He argued that “${\mathrm{SpO}}_{2}$ should not be taken at face value” and that the use of ${\mathrm{SpO}}_{2}$ “requires clinical wisdom and an appreciation for its limitations.”

Another trend that has emerged in recent years is that the global pandemic has led to an increase in market revenues due to an increase in the adoption of fingertip POs. The global PO market was estimated to be worth between USD 2.5 and 2.9 billion in 2022. It is forecast to grow to USD 3.19 billion by 2030, with the biomedical optics devices sector accounting for a significant proportion of this growth.^251^ According to our own analysis, the number of patents published in the periods 2020–2023 increased each year (2020: n=3304, 2021: n=3875, 2022: n=3988, 2023: n=4282; Google Patents, with the keyword “pulse oximeter” in the abstract).

The past few years have therefore been another period of significant interest and importance in the development and application of PO. Aoyagi was present at the inception of this development in the final years of his life, which came to an end on April 18, 2020. It can be reasonably assumed that his invention has saved the lives of numerous individuals during the COVID-19 pandemic and that it will continue to do so in the future.

Regarding the technical development of PO technology in recent years, the following developments are worthy of mention. In 2022, Jakachira et al.^252^ published the development of a novel polarization imaging-based PO technique to measure ${\mathrm{SpO}}_{2}$ with a single-wavelength (780 nm) radially polarized vector beam. Concerning the optimal methodology for acquiring and processing PPG signals (a topic of relevance to PO), Charlton et al. have addressed this issue.^253^ In 2023, Park et al.^254^ presented a non-contact dual-wavelength PO system, the first wireless sock POs (BabySat and DreamSock; Owlet, Inc., Lehi, Utah, United States) for smart home baby monitoring of ${\mathrm{SpO}}_{2}$ and pulse rate with FDA approval^255^ [Fig. 8(k)] and a wearable ring-type PO (BodiMetrics, Manhattan Beach, California, United States) with FDA approval^256^ were released, and a study demonstrated that three smartwatches with PO functionality (Apple Watch 8, Samsung Galaxy Watch 5, or Withings ScanWatch) were capable of reliably detecting hypoxemia (${\mathrm{SpO}}_{2}<90\%$).^257^ Furthermore, Mehmood et al.^258^ showed that a conventional smartphone can determine ${\mathrm{SpO}}_{2}$ and heart rate with an acceptable quality.

In the current year, 2024, the first “over-the-counter” fingertip PO (MightySat, Masimo) was cleared by the FDA for medical use without a prescription, and an over-the-counter wireless sock PO (Stork Vitals+, Masimo) for infants was also FDA-approved.^259^ This year, a prototype PO with a novel high-performance organic photodetector was developed,^260^ and a study showed a good agreement between ${\mathrm{SpO}}_{2}$ determination with an Apple Watch compared to medical-grade PO.^261^ However, another study showed that smartwatch-based PO devices, including the Apple Watch and Withings ScanWatch, exhibited insufficient reliability in detecting hypoxia in COVID-19 patients.^262^

## 4 Summary, Conclusions, and Outlook

In our historical review, we examined the evolution of oximetry in general and PO in particular. We traced the key stages of a long and fascinating story that has unfolded from the first half of the twentieth century to the present day. Without doubt, PO has become an integral part of modern medical care and has proven to be an important tool for physiological monitoring. As evidenced by the global COVID-19 pandemic, the clinical utility of PO has been demonstrated, yet the technology has also revealed limitations, particularly in relation to skin pigmentation bias. It seems probable that these issues will be resolved in the near future, paving the way for PO to continue its triumphant progress in both the medical field and in at-home applications for users seeking to monitor health.

## Data Availability

All data in support of the findings of this paper are available within the article.
